# Effect of multimodal exercise training on physical fitness indices, cognitive status, and depressive symptoms in Alzheimer's disease

**DOI:** 10.1590/1980-5764-DN-2022-0008

**Published:** 2023-05-29

**Authors:** Amir Hossein Haghighi, Masoud Barzoei, Seyed Alireza Hosseini Kakhak, Francesco Budini, Hadi Shahrabadi

**Affiliations:** 1 Hakim Sabzevari University, Faculty of Sport Sciences, Department of Exercise Physiology, Sabzevar, Iran.; 2 Ferdowsi University of Mashhad and Hakim Sabzevari University, Faculty of Sport Sciences, Department of Exercise Physiology, Mashhad and Sabzevar, Iran.; 3 University of Graz, Institute of Human Movement Science, Sport and Health, Graz, Austria.

**Keywords:** Exercise, Physical Fitness, Depression, Alzheimer Disease, Exercício Físico, Aptidão Física, Depressão, Doença de Alzheimer

## Abstract

In Alzheimer's disease (AD) patients, low levels of physical fitness (PF) and cognitive status are associated with high rates of depression. However, this condition can be improved through physical training.

**Objective:**

The aim of the present study was to investigate the effect of multimodal exercise training (MET) on aerobic endurance, muscular strength, agility, dynamic balance, cognitive status, and depressive symptoms in men with mild-to-moderate AD.

**Methods:**

A total of 25 elderly men with a diagnosis of mild-to-moderate AD were randomly categorized into an MET or a control group. The subjects in the MET group participated in a 12-week, three sessions per week MET program that included resistance, balance, and aerobic exercises. While the participants in the control group did not perform any regular exercise training during this period. Patients’ cognitive status and depressive symptoms were assessed by Mini-Mental State Examination and the Geriatric Depression Scale-15 (GDS-15) questionnaires. PF indicators such as aerobic endurance, muscular strength, agility, and dynamic balance, as well as cognitive status and depressive symptoms, were taken from all the subjects before and after MET.

**Results:**

The participants in the MET group improved handgrip, upper and lower body strength, agility, dynamic balance, and depressive symptoms (p<0.05). The intervention had no significant effect on aerobic endurance and cognitive status (p>0.05).

**Conclusions:**

MET is an effective strategy to improve muscular strength, agility, dynamic balance, and depressive symptoms in men with mild-to-moderate AD. It is recommended for AD patients to engage in this type of exercise to reduce AD complications.

## RESUMO.

Em pacientes com Alzheimer, baixos níveis de aptidão física (AF) e estado cognitivo estão associados a altas taxas de depressão. Essa condição, no entanto, pode ser melhorada através do treinamento físico.

**Objetivo:**

O objetivo do presente estudo foi investigar o efeito do treinamento multimodal (TMM) de resistência aeróbica, força muscular, agilidade, equilíbrio dinâmico, estado cognitivo e sintomas depressivos em homens com doença de Alzheimer (DA) leve a moderada.

**Métodos:**

25 homens idosos com diagnóstico de DA leve a moderada foram divididos aleatoriamente em um grupo TMM ou controle. Os indivíduos do grupo TMM participaram de um programa de TMM de 12 semanas, três sessões/semana, que incluía exercícios de resistência, equilíbrio e aeróbicos, enquanto os participantes do grupo controle não realizaram nenhum treinamento regular de exercícios durante esse período. O estado cognitivo e os sintomas depressivos dos pacientes foram avaliados pelos questionários *Mini-Mental State Examination* (MMSE) e *Geriatric Depression Scale-15* (GDS-15). Indicadores de AF, como resistência aeróbica, força muscular, agilidade e equilíbrio dinâmico, bem como estado cognitivo e sintomas depressivos, foram obtidos de todos os sujeitos antes e depois do TMM.

**Resultados:**

Os participantes do grupo TMM melhoraram a preensão manual e força de membros superiores e inferiores, agilidade, equilíbrio dinâmico e sintomas depressivos (p<0,05). A intervenção não teve efeito significativo na resistência aeróbica e no estado cognitivo (p>0,05).

**Conclusões:**

O TMM é uma estratégia eficaz para melhorar a força muscular, agilidade, equilíbrio dinâmico e sintomas depressivos em homens com DA leve a moderada. Recomenda-se que os pacientes com Alzheimer pratiquem esse tipo de exercício para reduzir as complicações da DA.

## INTRODUCTION

Alzheimer's disease (AD) is a progressive neurological disorder of the central nervous system characterized by profound impairment of cognitive function^1^. This disease is associated with the appearance of mental and behavioral disorders and reduced daily activities^2^. Decreased physical activity in the elderly leads to further progression of the disease and increases mortality^3^. Several studies have also reported decreased physical fitness (PF) and lower levels of muscle strength and agility in people with AD^3–5^. Regarding mental disorders, the prevalence of depression in AD patients is higher than that in healthy individuals^6,7^. In this regard, Huang et al.^8^, in their review study that compared the incidence rates of depression in old age between individuals with and without dementia, showed that subjects with dementia had a significantly higher risk for depression.

The high prevalence of AD and the high costs associated with patient care and treatment^9,10^ encourage the implementation of nonpharmacological therapies, including physical activity. Indeed, physical activity has been proposed to prevent AD and reduce its complications^11^. Physical activity facilitates neurogenesis and synaptogenesis, improves cerebral perfusion, maintains brain volume in regions vulnerable to AD, and reduces neuronal loss, β-amyloid accumulation, and tau phosphorylation^12^. Studies have shown that resistance training increases lower body strength, agility, and balance in AD patients^13^, and continuous and interval aerobic training improves aerobic fitness and functional capacities in the elderly with AD, but not cognitive performance^14^. Recently, the effectiveness of multimodal exercise training (MET) such as aerobic endurance, flexibility, resistance, balance exercises^15^, strength and balance exercises^16^, and muscle endurance, balance, flexibility, and aerobic exercises^17^ has been evaluated for the management of AD, showing encouraging results not only on physical parameters but also in terms of a decrease in the depression rate^17^. A review study by López-Ortiz et al.^18^ suggested that physical exercises) e.g., two or three weekly sessions of 30–60 min maintained for at least 2 months, combining both aerobic and muscle strengthening exercises) can improve some markers of cognitive function (at least for aerobic exercise) and physical function in patients with AD.

The abovementioned studies were mostly performed in patients with mild degree of AD^15,17^, and few studies have been performed on patients with mild-to-moderate AD^16^. As a result, there is no enough knowledge as to whether or not patients with mild-to-moderate degrees could also benefit from this approach. In addition, by reviewing previous studies, contradictory results were observed, especially in cognitive status of AD patients. In this regard, Parvin et al.^17^ showed that exercise training improves cognitive status, but Pedroso et al.^15^ found that this result was not confirmed by Salisbury and Yu^19^ and Enette et al.^14^

The aim of the present study was to answer the question of whether MET, including resistance training, balance, and aerobics, can have a significant impact on aerobic endurance, muscular strength, agility, dynamic balance, cognitive status, and depressive symptoms in men with mild-to-moderate AD. Considering the benefits of using combination exercises in previous studies^15–17^, we hypothesize that MET would improve aerobic endurance, muscular strength, agility, dynamic balance, and depressive symptoms in men with mild-to-moderate AD.

## METHODS

### Sample size

The number of subjects was calculated using G*Power (version 3.1.9.4) with an alpha coefficient of 0.05, statistical power of 0.80, and effect size of 0.59, based on previous research investigating the effects of dual-task training on cognitive status, physical performance, and brain waves of patients with AD^17^.

### Participants

**Figure 1:**
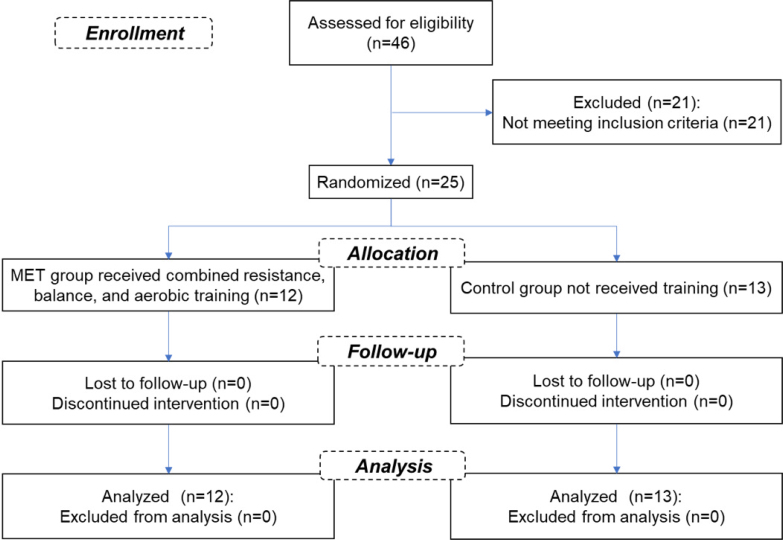
Flowchart of the study design.

Abbreviation: MET: multimodal exercise training.

A total of 46 patients with a diagnosis of AD who were staying in Hazrat Abolfazl Al-Abbas geriatric nursing home of Sabzevar, Iran, accepted to volunteer in the study. After assessment for eligibility (see inclusion/exclusion criteria below), 25 participants were classified into two groups: MET and control (Figure 1) using a simple random method (lottery), which was concealed until interventions were assigned (i.e., the patients were unaware of which group they would be allocated to, at the time they give their consent). In addition, the allocation of patients into two groups was done by researchers who were blinded to the characteristics of the patients.

#### Inclusion criteria

- Age of 60 years or older;
- Diagnosis of mild-to-moderate AD determined by a specialist physician;
- The ability to perform exercise training;
- Not participating in any regular exercise program in the last 6 months.

#### Exclusion criteria

- Incidence of heart disease;
- The presence of sports injuries during the training period;
- The unwillingness of the subject to complete the training program.

This study was approved by the committee of ethics of Hakim Sabzevari University, Sabzevar, Iran (IR.HSU.REC.1398.003). All ethical considerations were considered in this study, including obtaining consent to participate in the research, confidentiality of participants’ personal information, and familiarity with the purpose of the research and training protocol.

### Measurements

**Figure 2:**
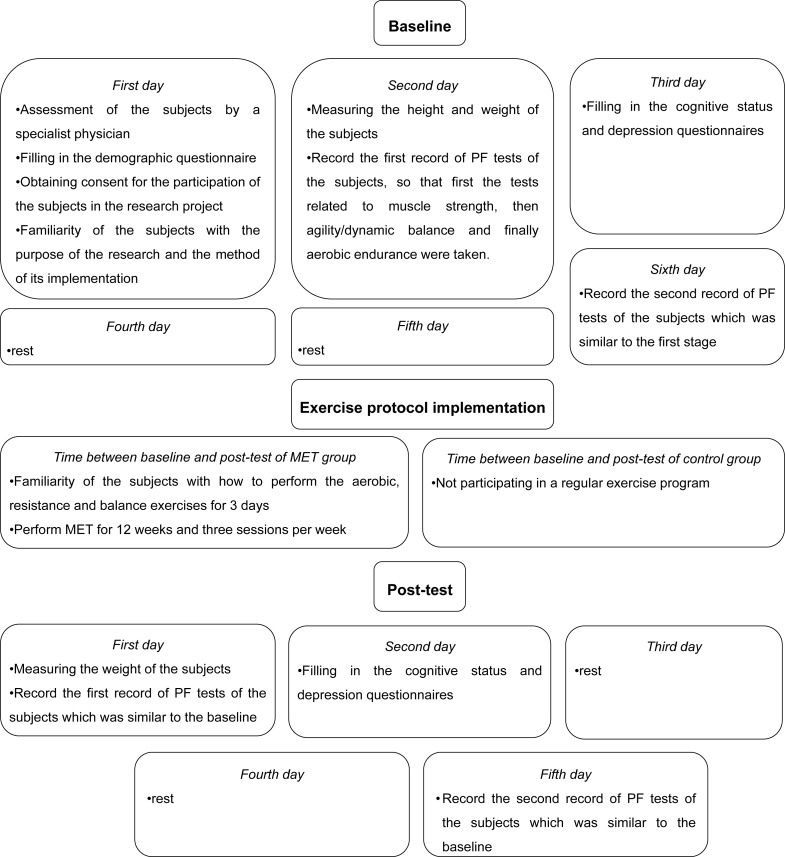
Scheduling of research implementation stages.

Abbreviations: MET: Multimodal Exercise Training; PF: physical fitness.

Data of subjects in the pre-test and post-test stages were collected by two experienced assessors according to a specific schedule (Figure 2). Before filling in the demographic questionnaires, cognitive status, and depressive symptoms and performing PF tests, explanations were given to acquaint the participants with the questions in the questionnaire and how to perform PF tests. PF tests were taken from the subjects in two stages with an interval of 3 days, and then the best record was considered for them. All tools used to collect data were appropriate to the condition of these individuals, and all safety considerations were considered during the execution of tests. The subjects warmed up for 10 min before starting the PF tests. We used Rikli and Jones's study^20^ to measure body mass index (BMI), aerobic endurance, upper and lower body strength, and agility/dynamic balance and Fritz et al. study^21^ to measure handgrip strength. A stopwatch (Model DS-013, Q&Q, China) was used to record time.

### Anthropometric

To measure standing height and body weight, a SECA stadiometer with an accuracy of 1 mm and a portable SECA scale with an accuracy of 0.1 kg (Seca770, Hamburg, Germany) were used, respectively. Body composition was also assessed using BMI (weight/height^2^).

### Aerobic endurance

A 6-min walk test was used to estimate the subjects’ aerobic endurance. The longest distance a person could cover in 6 min without assistance was his record.

### Handgrip strength

Handgrip dynamometer (Model SH5001, Saehan, South Korea) was used to estimate handgrip strength. First, the person sits on a chair, puts his hand on the handle of the chair, and then presses the handle of the handgrip dynamometer so that the arms are in line with the body and the elbow angle is ≈90°. The maximum force recorded by the dynamometer in kilograms was considered his record.

### Upper body strength

The 30-s arm curl test was used to estimate upper body strength. The weight of the dumbbell for elderly men was 3.63 kg. The number of movements performed by the subject in a period of 30 s was considered a record.

### Lower body strength

To estimate this item, the 30-s chair stand test with arms folded across the chest was used so that the height of the chair is 43 cm. The number of full stands in 30 s was considered a record.

### Agility/dynamic balance

The 8-foot up-and-go test was used to measure the subjects’ agility/dynamic balance. The time spent to get up from a seated position, walk 8 feet, turn, and return to a seated position on a chair was considered a record.

### Cognitive status

The Mini-Mental State Examination (MMSE) questionnaire was used to assess cognitive status. This questionnaire is used as a practical test to measure cognitive functions. This questionnaire, prepared by Folstein et al. in 1975, includes categories of orientation to time, orientation to place, repetition and registration, attention and calculation, recall, naming, repetition, comprehension, reading, writing, and drawing.^22^ The maximum total score is 30; a score of 27–30, 20–26, 10–19, and <10 is considered normal, mild, moderate, and severe AD, respectively^23^. The MMSE questionnaire used in this study in Iran has been standardized by Saidyan et al.^24^ The researchers confirmed the discriminant validity through an independent samples t-test (p<0.001). They also used Cronbach's alpha coefficient to determine the reliability of the questionnaire, which was estimated to be 0.81.

### Depressive symptoms

Depressive symptoms were measured by the Geriatric Depression Scale-15 (GDS-15). This questionnaire was prepared by Sheikh and Yesavage. It is a suitable test for diagnosing depressive symptoms in the elderly. This questionnaire consists of 15 questions, and each question is answered yes or no. Questions 1, 5, 7, 11, and 13 are scored in reverse. The range of scores is between 0 and 15^25^. The GDS-15 questionnaire used in this study in Iran has been standardized by Malakouti et al.^26^ so that these researchers confirmed the validity of differentiation for this questionnaire (p<0.001). The reliability of this questionnaire was also reported in the Iranian aging community through Cronbach's alpha, split half, and test-retest of 0.9, 0.89, and 0.58, respectively.

### Intervention

The training progression is summarized in Table 1. The experimental group performed the MET for 12 weeks, three sessions per week (Monday, Wednesday, and Saturday) from 10:00 a.m. to 11:30 am at the geriatric nursing home of Sabzevar (in Hazrat Abolfazl Al-Abbas, Iran). The training program consisted of three parts, namely, resistance training, balance training, and aerobic training, each preceded by a 10-min warm-up and followed by a 10-min cool-down. The training always started with resistance training that included squats, chest presses, shoulder presses, and leg extension, while holding a 1-kg medical ball. The balance training part included four exercises:

- Standing up from the chair and walking 2 m while raising the thigh and touching it with the hand;
- Walking 2 m of tandem walk (thumb to heel);
- Performing the modified bird-dog movement; and
- Lifting one leg and standing on the support leg.

**Table 1:** Exercise protocol of the multimodal exercise training group.

Exercises training		Weeks			
		1–3	4–6	7–9	10–12
Resistance training	Sets (n)	3	4	5	6
	Repeats (n)	8–10	8–10	8–10	8–10
	Rest between sets (min)	1–2	1–2	1–2	1–2
Balance exercises	Sets (n)	2	3	4	5
	Rest between circles (min)	1	1	1	1
Aerobic exercise	Intensity (%HR_max_/RPE)	70/12	70/12	75/14	75/14
	Sets×time (n×min)	3×2	3×2	4×2	4×2
	Rest between sets (min)	1–1.5	1–1.5	1–1.5	1–1.5

Abbreviations: RPE: Rate of Perceived Effort; %HR max: the percentage of the maximum heart rate.

The final stage of the training program is moderate-intensity aerobic exercise, which includes brisk walking, moving to the sides, and performing rhythmic movements with music. Verbal encouragement was used during the implementation of the training protocol to increase the motivation of the elderly. The intensity of exercise was also monitored through the Borg Rating of Perceived Exertion^27^ for the elderly who used beta-blockers or other medication that prevented the increase in heart rate and through a heart rate monitor watch (model PM100, Germany) for other elderly. The control group did not perform any regular exercise training during the study period.

### Statistical analysis

Data were checked for normality using the Shapiro-Wilk test. Mean and standard deviation indices were used to report data with normal distribution, and median and quarter indices were used for data with abnormal distribution. Levine's test was also used to determine the homogeneity of variances. Independent-samples t, Mann-Whitney U, and analysis of covariance (ANCOVA) tests were used to compare the variables between groups. The paired-samples t-test and Wilcoxon test were used to compare the variables within the group. Data were analyzed using the SPSS version 25 software with a significance level of less than 0.05.

## RESULTS

No significant difference was observed for age and anthropometric values between MET and control groups in the baseline (Table 2).

**Table 2:** Demographic information of men with Alzheimer's disease (n=25).

Variables	Groups		
	MET (n=12)	Control (n=13)	p-value
Age (years), mean±standard deviation	69.92±8.16	72.92±6.17	0.307*
Height (cm), mean±standard deviation	163.33±5.84	166.15±3.67	0.158*
Weight (kg), median (Q1–Q3)	59.05 (53.37–62.15)	56.20 (54.20–58.05)	0.384†
BMI (kg/m^2^), median (Q1–Q3)	21.12 (19.70–24.24)	20.08 (19.25–22.02)	0.192†

Abbreviations: MET: Multimodal Exercise Training; BMI: body mass index.

* Notes: Independent samples t-test;

† Mann-Whitney U test.

As reported in Table 3, the present study showed that handgrip and upper body and lower body strength were significantly increased in men with mild-to-moderate AD. After performing the training protocol, the rate of increase in handgrip, upper body, and lower body strength in the MET group was 4.03% (z=-1.651, p=0.099, r=0.477), 17.27% (t=-4.710, p=0.001, r=0.818), and 10.10% (t=-1.890, p=0.085, r=0.495), respectively. In the control group, the strength of the handgrip increased by 0.98% (t=-1.897, p=0.082, r=0.466), but the strength of the upper and lower body decreased by 3.96% (z=-0.447, p=0.655, r=0.124) and 2.40% (t=1.477, p=0.165, r=0.392), respectively.

**Table 3:** Comparison between groups of physical fitness indices, Mini-Mental State Examination and Geriatric Depression Scale-15 in men with Alzheimer's disease.

	Groups	Baseline			Post-test			Statistical result		
Aerobic endurance (m), mean±standard deviation			Min	Max		Min	Max	F	p-value	pη^2^
	MET	195.22±62.95	100.00	275.13	199.01±67.53	100.00	288.37	1.558	0.225	0.066
	Control	141.13±32.73	107.90	211.87	138.09±31.87	108.00	211.17			
Handgrip strength (kg), median (Q1–Q3)								z	p-value	r
	MET	11.00 (10.00–14.50)	9.00	22.00	12.50 (12.00–14.75)	10.00	16.00	−2.959	0.003*	0.592
	Control	9.00 (7.50–11.50)	7.00	13.00	9.00 (7.00–11.50)	6.00	13.00			
Upper body strength (n), mean±standard deviation								F	p-value	pη^2^
	MET	9.17±2.95	6.00	14.00	10.75±2.80	6.00	16.00	21.054	<0.001†	0.489
	Control	7.85±1.57	6.00	11.00	7.92±1.44	6.00	10.00			
Lower body strength (n), mean±standard deviation								F	p-value	pη^2^
	MET	8.25±2.01	6.00	12.00	9.08±2.35	6.00	13.00	6.932	0.015‡	0.240
	Control	7.77±1.01	6.00	10.00	7.46±1.20	6.00	10.00			
Agility/dynamic balance (s), median (Q1–Q3)								z	p-value	r
	MET	16.72 (13.44–20.88)	9.35	25.00	16.19 (11.00–21.90)	9.28	25.00	-2.068	0.039‡	0.414
	Control	21.89 (18.75–23.87)	16.43	26.10	22.34 (19.10–24.63)	15.87	27.02			
MMSE, median (Q1–Q3)								z	p-value	r
	MET	18.00 (18.00–20.75)	18.00	23.00	19 (19.00–20.00)	17.00	22.00	-0.978	0.328	0.196
	Control	20.00 (18.50–22.00)	18.00	23.00	20.00 (18.00–21.50)	17.00	24.00			
GDS-15, mean±standard deviation								F	p-value	pη^2^
	MET	5.17±1.53	2.00	8.00	4.75±1.55	2.00	8.00	4.403	0.048‡	0.167
	Control	5.39±1.12	4.00	8.00	5.54±1.05	4.00	7.00			

Abbreviations: MET: Multimodal Exercise Training; MMSE: Mini-Mental State Examination; GDS-15: Geriatric Depression Scale-15; Notes: r and pη^2^: effect size; F-values based on ANCOVA; z-values based on Mann-Whitney U test.

* Difference between groups is significant at 0.01;

† Difference between groups is significant at 0.001;

‡ Difference between groups is significant at 0.05.

Despite a 1.94% increase in the 6-min walk test of the MET group (t=-1.842, p=0.093, r=0.485) and a 2.15% decrease in the control group (z=-0.941, p=0.347, r=0.261), the 12-week MET on the aerobic endurance index in men with mild-to-moderate AD did not change significantly.

The findings of the present study showed that the implementation of a 12-week MET had a significant effect on the agility/dynamic balance index in men with mild-to-moderate AD. This type of exercise reduced the 8-feet up-and-go time by 4.27% in the MET group (t=1.167, p=0.268, r=0.332), while this variable increased by 2.14% in the control group (t=-2.151, p=0.053, r=0.527).

During the 12 weeks of the MET, there was no significant difference in the cognitive status of men with mild-to-moderate AD between the two groups. We observed an increase of 0.87% (z=-0.577, p=0.564, r=0.167) and a decrease of 1.14% (t=1.148, p=0.273, r=0.315) in the MET and control groups, respectively.

Our findings showed that the implementation of a 12-week MET had significant effect on the depressive symptoms in men with mild-to-moderate AD. This type of exercise decreased the depressive symptoms by 8.12% in the MET group (t=2.803, p=0.017, r=0.645), while this variable increased by 2.78% in the control group (z=-0.632, p=0.527, r=0.175).

## DISCUSSION

The present study examined the effects of an MET course on aerobic endurance, muscular strength, agility, dynamic balance, cognitive status, and depressive symptoms in men with mild-to-moderate AD.

We did not observe a significant change in aerobic endurance, which is in agreement with the results of Pedroso et al.^15^ and in disagreement with the results of Parvin et al.^17^ and Enette et al.^14^, Pedroso et al.^15^ stated that due to the performance of functional task training, no significant change in the distance of the 6-min walk test was observed. The researchers said that the increase in aerobic endurance index was lower than expected, which can be attributed to a lack of motivation or fatigue during the performance of functional task exercises in the elderly. These factors also prevent subjects from exercising at the expected level of heart rate, so the researcher does not achieve the goals of his exercise program. If the aim of the study is to improve aerobic endurance, it is better to increase the duration of aerobic exercise in each session as well as the length of the training period. To achieve this goal, it is necessary to design an exercise training program in such a way that this group of people in the community do not get tired during the exercise due to their problems.

MET led to improvements in handgrip and upper body and lower body strength. The results of the present study are in line with the results of Garuffi et al.^13^, Chang et al.^28^, Pedroso et al.^15^, Parvin et al.^17^, and Cezar et al.^16^, are in contradiction with the results of Sobol et al.^29^, Garuffi et al.^13^ showed that 16 weeks of resistance training leads to a significant increase in the lower body strength of elderly with AD. In this regard, Chang et al.^28^ showed that 12 weeks of resistance increases handgrip strength in AD patients with sarcopenia. Pedroso et al.^15^ also stated that performing 12 weeks of functional task training improved upper body strength in the elderly with AD, but not lower body strength. In contrast, Sobol et al.^29^ stated that 16 weeks of aerobic exercise did not significantly alter lower body strength in AD patients. By examining the results of this study, a greater increase in muscle strength was observed in the control group than in the aerobic exercise group, which may be due to the design of the exercise program in this study.

We observe an improvement in agility and dynamic balance, which is in agreement with the results of Garuffi et al.^13^ and Parvin et al.^17^ and in disagreement with the results of Pedroso et al.^15^ and Cezar et al.^16^, Garuffi et al.^13^ stated that as a result of resistance training after 16 weeks, there was a significant increase in the agility and balance of the elderly with AD. In contrast, Pedroso et al.^15^ stated that the agility index did not change significantly after performing functional task training for 12 weeks. Due to the significant reduction of agility in the control group, they stated that exercise could only prevent the reduction of agility in the elderly with AD. The agility/dynamic balance test in our study increased the lower body strength of the elderly, and this increase in strength may have affected agility. Increasing neuromuscular coordination and neural adaptation of the muscular spindles and Golgi tendon organs also improve agility.^30^

We did not observe a significant change in cognitive status. Our results are in agreement with the results of Pedroso et al.^15^, Salisbury and Yu^19^, and Enette et al.^14^, and in disagreement with the results of Parvin et al.^17^, Pedroso et al.^15^ did not observe a significant increase in MMSE score during 12 weeks of functional task training. The authors suggested that the process of cognitive decline increases during the period of aging and may prevent the effectiveness of exercise.^15^ Salisbury and Yu^19^ examined changes in aerobic fitness and cognitive status after exercise in AD patients. Although cognitive status did not improve in this study, the results showed that there is a direct relationship between aerobic fitness and cognitive status among these patients so that with increasing aerobic fitness, the cognitive status improves in this group of people.

The effect of MET on depressive symptoms in AD patients has already been investigated by others before us.^17,28,31–33^ In agreement with previous works,^17,28,31–33^ our results confirm that the depressive symptoms in these patients can be significantly reduced. Yu et al.^31^ investigated the effect of aerobic exercise on depression in AD patients. The results showed that the rate of depression in these patients was significantly reduced. Although depressive symptoms were reduced in this study, the authors stated that these changes were not clinically significant because subjects had low depressive symptoms at baseline and post-test stage. Chang et al.^28^ examined the effect of resistance exercise on depression in mild AD patients with sarcopenia. The results showed that the rate of depression in this group of people has decreased significantly. Adam et al.^32^ investigated the effect of combined dance and relaxation exercises on reducing depression in cognitively impaired elderly. The results showed that the rate of depression in the elderly was significantly reduced. Chen et al.^33^ examined the effect of 15 months of resistance band training on depression in the elderly with dementia. The results showed that depression in this group of people has decreased. Exercising with music and creating an enjoyable and intimate environment may help reduce depression in men with AD.

### Limitations

This study has several limitations that may affect the generalization of the results to the Alzheimer's community. The most important limitations of this study include the lack of precise control of activities outside the MET program of the subjects and the lack of control their mental states. Another limitation of the research was the use of MMSE and GDS-15 to evaluate cognitive status and depressive symptoms, respectively. It is better to use questionnaires in future studies that are more in line with the research objectives and provide more accurate and practical information. Not registering the protocol in the clinical trial and insufficient demographic information of the participants (e.g., medication, economic status, living situation, years of disease, and treatment) are among the limitations of the research.

The results of the study showed that performing a mix of strength, balance, and aerobic exercise for 12 weeks with three sessions per week can lead to a significant improvement in muscular strength, agility, dynamic balance, and depressive symptoms in the elderly, but no significant effect on aerobic endurance and cognitive status. It is recommended that regular exercise training be done in geriatric nursing homes to reduce complications of mild-to-moderate AD.
